# Ser9p-GSK3β Modulation Contributes to the Protective Effects of Vitamin C in Neuroinflammation

**DOI:** 10.3390/nu16081121

**Published:** 2024-04-10

**Authors:** Melania Ruggiero, Antonia Cianciulli, Rosa Calvello, Chiara Porro, Francesco De Nuccio, Marianna Kashyrina, Alessandro Miraglia, Dario Domenico Lofrumento, Maria Antonietta Panaro

**Affiliations:** 1 Department of Biosciences, Biotechnologies and Environment, University of Bari, 70125 Bari, Italy; melania.ruggiero@uniba.it (M.R.); antonia.cianciulli@uniba.it (A.C.); rosa.calvello@uniba.it (R.C.); mariaantonietta.panaro@uniba.it (M.A.P.); 2 Department of Clinical and Experimental Medicine, University of Foggia, 71100 Foggia, Italy; 3 Department of Biological and Environmental Sciences and Technologies, Section of Human Anatomy, University of Salento, 73100 Lecce, Italy; francesco.denuccio@unisalento.it (F.D.N.); marianna.kashyrina@unisalento.it (M.K.); alessandro.miraglia@unisalento.it (A.M.); dario.lofrumento@unisalento.it (D.D.L.)

**Keywords:** microglia, GSK3β, Wnt/β-catenin signaling pathway, neuroinflammation, neuroprotection, Vitamin C

## Abstract

Background. The prolonged activation of microglia and excessive production of pro-inflammatory cytokines can lead to chronic neuroinflammation, which is an important pathological feature of Parkinson’s disease (PD). We have previously reported the protective effect of Vitamin C (Vit C) on a mouse model of PD. However, its effect on microglial functions in neuroinflammation remains to be clarified. Glycogen synthase kinase 3β (GSK3β) is a serine/threonine kinase having a role in driving inflammatory responses, making GSK3β inhibitors a promising target for anti-inflammatory research. Methods. In this study, we investigated the possible involvement of GSK3β in Vit C neuroprotective effects by using a well-known 1-methyl-4-phenyl-1,2,3,6-tetrahydropyridine (MPTP)-induced animal model of PD and a cellular model of neuroinflammation, represented by Lipopolysaccharide (LPS)-activated BV-2 microglial cells. Results. We demonstrated the ability of Vit C to decrease the expression of different mediators involved in the inflammatory responses, such as TLR4, p-IKBα, and the phosphorylated forms of p38 and AKT. In addition, we demonstrated for the first time that Vit C promotes the GSK3β inhibition by stimulating its phosphorylation at Ser9. Conclusion. This study evidenced that Vit C exerts an anti-inflammatory function in microglia, promoting the upregulation of the M2 phenotype through the activation of the Wnt/β-catenin signaling pathway.

## 1. Introduction

Neuroinflammation is an immune response finalized to preserve the integrity of central nervous system (CNS) parenchyma following pathogenic stimuli and tissue injury [1]. Microglia, being the sentinel immune cells of the brain, constitute the first line of defense and react to detrimental stimuli, switching from a resting state to the activated M1 phenotype to initiate the inflammatory response. After the removal of the inflammatory stimuli, microglia switch to an alternative active state, the M2 phenotype, promoting debris clearance and reducing inflammation. Although the inflammatory response is essential to neutralize potentially harmful agents, a prolonged or chronic inflammation drives an overabundance of inflammatory cytokines and neurotoxic factors, fostering the M1 phenotype and, in turn, the release of additional pro-inflammatory cytokines, contributing to the amplification of the neuroinflammation [2].

Parkinson’s disease (PD) is the second most common neurodegenerative disease, characterized by progressive loss of dopaminergic neurons within the Substantia Nigra (SN), with the consequent depletion in dopamine in the striatum and accumulation of α-synuclein protein [3]. The aggregation of α-synuclein promotes neuroinflammation, which eventually contributes to the pathogenesis of PD [4,5].

Glycogen synthase kinase-3 (GSK3) β is a serine/threonine kinase widely present in the adult brain, in contrast to the GSK3α isoform, which is highly expressed only in cerebral cortex, hippocampus, striatum, and Purkinje cells of the cerebellum [6]. Because of its wide distribution, the attention has been focused on GSK3β, which is normally found in a constitutively active state achieved with the autophosphorylation of Tyr216 residue. The inhibition of GSK3β is due to the phosphorylation of Ser9 residue via the activation of Akt and p38 MAPK, among others [7]. GSK3β plays a role in regulating immune responses, since its activated state seems to be associated with pro-inflammatory responses, whereas the inactivated form seems to exert anti-inflammatory effects [8]. In this regard, GSK3β dysregulation has been linked to PD pathogenesis, its overactivation being associated with neuroinflammation and dopaminergic neuron death [9].

Vitamin C (Vit C), also known as ascorbic acid or ascorbate, is a water-soluble vitamin well known to have both antioxidant and anti-inflammatory properties [10], making it a potential candidate for counteracting the progression of neurodegenerative diseases. Although its role in reducing neuroinflammation and oxidative stress has been elucidated, the molecular mechanism underlying these properties is not yet clear. In our previous work [11], we demonstrated the Vit C ability to reduce neuroinflammation by modulating microglial and astrocyte activation, reducing the expression of pro-inflammatory mediators, as well as ameliorating gait and spontaneous locomotor activity in a 1-methyl-4-phenyl-1,2,3,6-tetrahydropyridine (MPTP) mouse model of PD. 

To investigate the molecular mechanisms by which Vit C exerts its anti-inflammatory and neuroprotective effects, in this study, we examined the involvement of GSK3β after Vit C treatment both in a MPTP mouse model of PD and in an in vitro cellular model of neuroinflammation, represented by LPS-activated BV-2 microglial cells. 

## 2. Materials and Methods

### 2.1. Cell Culture and Treatments

The immortalized BV-2 mouse microglial cell line (ICLCATL03001) was purchased from Interlab Cell Line Collection (Banca Biologica e Cell Factory, Genoa, Italy) and was grown in high-glucose Dulbecco’s Modified Eagle Medium (DMEM) supplemented with 10% Fetal Bovine Serum (FBS), 1% Penicillin–Streptomycin (Pen-Strep) Solution (Gibco-Thermo Fisher Scientific, Waltham, MA, USA), and 1% Glutamine (Sigma-Aldrich, St. Louis, MO, USA) at 37 °C in a humidified incubator set to 5% ${CO}_{2}$. Once reaching a confluence of 80%, cells were washed with Phosphate-Buffered Saline (PBS) (Sigma-Aldrich, St. Louis, MO, USA), trypsinized with Trypsin–EDTA (0.25%) (Gibco-Thermo Fisher Scientific, MA, USA), and collected to be seeded in 6-well plates, T25 flasks, and 96-multiwell plates, respectively, for a Western blot, RT-PCR, and MTT assay. For the in vitro treatments, three different concentrations of Vit C (Sigma-Aldrich, St. Louis, MO, USA), ranging from 10 ng/mL to 100 ng/mL, alone or in combination with LPS (1 μg/mL) (Sigma-Aldrich) were tested. In addition to the untreated group (C), BV-2 cells were submitted to a combined treatment with Vit C, ranging from 10 ng/mL to 100 ng/mL, for 24 h, followed by 1 μg/mL LPS and then harvested after 48 h. Since in the combined treatment BV-2 cells were exposed to Vit C for 72 h and to LPS for 48 h, two groups of cells were treated, respectively, with Vit C for 72 h and with LPS for 48 h. Lastly, in another experimental set, to assay the p38 inhibition, cells were treated with SB203580 (Sigma-Aldrich, St. Louis, MO, USA), a selective inhibitor of p38 MAPK, 1 h before the onset of the treatments described above. 

### 2.2. Animals and Treatment Protocols

For the in vivo study, we used overall 48 male mice of the 129 SV strain weighing between 22 g and 26 g (Envigo, Udine, Italy). Complying with the protocols approved by the Ministry of Health-Section VI: Animal welfare and in accordance with the Institutional Animal Committee and in accordance with the European Union (EU) Directive 2010/63/EU for animal experiments, mice were divided equally in four groups and subjected to different treatments.

The control group was treated with water for 10 days by intragastric gavage and the second group with Vit C (15 mg/Kg) (Sigma-Aldrich, St. Louis, MO, USA) by intragastric gavage daily for 10 days; twelve mice were treated with water for 10 days and received four doses of MPTP (20 mg/Kg) at 2 h intervals by intraperitoneal injection on the 3rd day; lastly, the fourth group was stimulated with Vit C daily for 10 days and received four doses of MPTP at 2 h intervals on the 3rd day. At the end of treatments, mice were anesthetized by the inhalation of 4% isoflurane and sacrificed by cervical dislocation [12].

### 2.3. Immunohistochemistry

Sections of 10 μm, obtained from paraffin-embedded brains, were incubated with a 1:1500 solution of anti-TH mouse MoAb (BioLegend, Amsterdam, The Netherlands) overnight. The following day, incubations were performed with a biotinylated secondary antibody and then with extravidin–peroxidase. Subsequently, using the chromogenic substrate 3,3′-diaminobenzidine (DAB), a brown-stained oxidation product was visualized in correspondence with immunocomplexes. Images were acquired with a DS-5M digital camera assembled on a Nikon Eclipse E800 microscope (Nikon Instruments S.p.A, Campi Bisenzio, Florence, Italy).

### 2.4. MTT Assay

The MTT assay was used to measure cell metabolic activity and therefore cell viability. At the end of the treatments described above, cells were incubated with 3-(4,5-Dimethylthiazol-2-yl)-2,5-diphenyltetrazolium bromide (MTT) (Sigma-Aldrich, St. Louis, MO, USA) at a working concentration of 1 mg/mL in PBS for 4 h at 37 °C in a humidified atmosphere with 5% ${CO}_{2}$ to allow the conversion of tetrazolium in formazan. Insoluble formazan crystals were solubilized in 150 μL of Dimethylsulfoxide (DMSO) for 20 min under stirring. Cell viability was measured by reading the absorbance at 540 nm on Cytation 3 Cell Imaging Multi-Mode Reader (Biotek, Winooski, VT, USA). Values were converted to percentages taking the control as 100% and expressed as the average percentage ± SD. 

### 2.5. Protein Extraction and Immunoblotting

Protein extraction was performed both from Substantia Nigra pars compacta (SNpc) and BV-2 cell cultures. After tissue isolation, SNpc was homogenized by adding a RIPA buffer and performing multiple freezing and thawing cycles. At the end, the lysates were centrifuged at 13,000× *g* for 20 min to remove cell debris. 

BV-2 cells were washed with PBS, detached from the plate by using a cell scraper, and collected by spinning at 4 °C at 600× *g* for 10 min. After centrifugation, cells were lysed in a RIPA buffer consisting of Tris (50 mM) (PH 8), 1.5 M NaCl, 1% Triton-X, and 0.1% SDS; improved with PMSF (100 μM), Leupeptin (1 µM), and Aprotinin (4 U/mL) protease inhibitors; and submitted to multiple freezing and thawing cycles to improve lysis. The protein concentration was determined by the Bradford Assay and an amount of 20 μg was loaded on 4–12% precast polyacrylamide gels (Thermo Fisher Scientific Inc., MA, USA) with NuPAGE™ LDS Sample Buffer (4X) and NuPAGE™ Sample Reducing Agent (10X) (Thermo Fisher Scientific Inc., MA, USA) to be separated by SDS-PAGE. Proteins were transferred on a nitrocellulose membrane, blocked with 5% (*w*/*v*) non-fat dried milk for 1 h, and then washed 3 times with 0.1% Tween 20–PBS (T-PBS). Then, following a standard avidin–biotin complex procedure, membranes were incubated in the dark with mouse monoclonal antibody (moAb) anti-β-actin (sc-47778), mouse moAb anti-p-GSK3β (Ser9) (sc-373800), mouse moAb anti-GSK3β (sc-53931), rabbit polyclonal antibody (poAb) anti-p-AKT1/2/3 (Ser473) (sc-514032), mouse moAb anti-AKT (sc-377457), rabbit poAb anti-p-p38 MAPK (sc-7975-R), mouse moAb anti-p38 (sc-7972), mouse moAb anti-p-IKBα (Ser32) (sc-8404), mouse moAb anti-IKBα (sc-4094), rabbit poAb anti-TLR4 (sc-10741), rabbit poAb anti-SOD1 (sc-11407), and mouse moAb anti-NRF2 (sc-365949) (all from Santa Cruz Biotechnology, Inc., Milan, Italy) at a 1:500 dilution. In the end, membranes were incubated with horseradish peroxidase (HRP)-conjugated secondary antibodies (Santa Cruz Biotechnology), diluted 1:10,000, for 60 min at room temperature in the dark on a shaker. After three washes with 0.1% T-PBS, immunoreactive bands were acquired by ChemiDoc XRS+ Imager (Bio-Rad Laboratories, Inc., Hercules, CA, USA) and the optical density of bands normalized with β-actin was quantified to be expressed as the mean ± SD. 

### 2.6. RNA Isolation and RT-PCR

Total RNA was isolated by using GenElute^™^ Mammalian Total RNA Miniprep Kit (Sigma-Aldrich Corporation, MO, USA) according to the manufacturer’s instructions. RNA was reverse-transcribed back into cDNA with SuperScript III Reverse Transcriptase (Thermo Fisher Scientific) and target cDNAs were amplified with a 2720 Thermal Cycler (Applied Biosystems, Waltham, MA, USA). Table 1 reports the cDNA targets and the primers used for PCR. Then, samples were loaded on the agarose gel together with TriTrack DNA Loading Dye (6X) (Thermo Fisher Scientific Inc., Waltham, MA, USA) to visualize DNA bands upon UV illumination. The mean value of band intensity was measured by using ImageJ software (version 2.14.0), normalized to GAPDH, and expressed as the mean ± SD.

### 2.7. Immunofluorescence

A total number of 2.5 × 10^3^ cells/well was seeded in a 24-multiwell plate containing round sterile coverslips and treated following the experimental design described above. After 48 h, cells were washed with PBS and fixed with paraformaldehyde (PFA), 4%, for 15 min at room temperature. Once the fixation agent was removed with PBS rinses, the permeabilization was performed with PBS supplemented with 0.1% Triton X-100 for 10 min at 37 °C. Cells were washed and blocked with goat serum (10%) (Sigma Aldrich, St. Louis, MO, USA) for 45 min at 37 °C. At the end of the blocking step, specimens were incubated with mouse moAb anti-β catenin (Santa Cruz Biotechnology, Inc., Milan, Italy) diluted 1:100 in goat serum, 1.5%, overnight at 4 °C to allow the immunobinding. After rinsing with PBS, anti-β catenin was stained with a secondary antibody goat anti-mouse IgG (Fc-specific)-FITC (Sigma Aldrich, St. Louis, MO, USA) diluted 1:200 in goat serum, 1.5%, for 1 h at room temperature. In the end, cells were counterstained with DAPI (Sigma Aldrich, St. Louis, MO, USA) at a working concentration of 1 μg/mL for 30 min at 37 °C. Coverslips were mounted onto microscope slides for microscopy observation with a P-phenylenediamine (PPD) antifade mounting solution containing 20 mg of PPD powder (Sigma Aldrich, St. Louis, MO, USA), 10% 1 M Tris (PH 9), and 70% glycerol. Fluorescent images were acquired with two fluorescent channels (498/517 nm for FITC and 360/463 nm for DAPI) on an inverted Leica LSM TSC SP2 AOBS confocal microscope provided with a 63× oil-immersion objective. 

### 2.8. Cell Image Processing and Morphometric Analysis

BV-2 cells were observed at 200X magnification with an inverted microscope equipped with a high-definition camera (Eclipse TS100, Nikon Instruments S.p.A, CampiBisenzio, Florence, Italy). Image processing was performed by ImageJ software. For the morphometric analysis, RGB images were preferentially used. Briefly, cell images were converted to 8 bits, normalized by background subtraction, adding a scale bar of 10 pixel/μm. After freehand marking, cells were analyzed by the measuring area, perimeter, circularity, and aspect ratio (AR). 

### 2.9. Statistical Analysis

The significance of immunoblotting and RT-PCR data was assessed with RStudio software, version 4.3.1 (R Foundation for Statistical Computing, Vienna, Austria). Once verified using the ANOVA assumptions with Shapiro–Wilk and Bartlett tests, data were analyzed with the one-way ANOVA followed by the post hoc Tukey test, assuming only *p*-values < 0.05 as significant. Concerning the morphometric analysis, Statgraphics Centurion (Statgraphics Technologies Inc., The Plains, VA, USA) was used for carrying out the analysis of variance (one-way ANOVA) and Tukey’s post hoc test. Values of *p* < 0.05 were considered statistically significant.

## 3. Results

### 3.1. Vit C Effects on BV-2 Cell Viability

**Figure 1:**
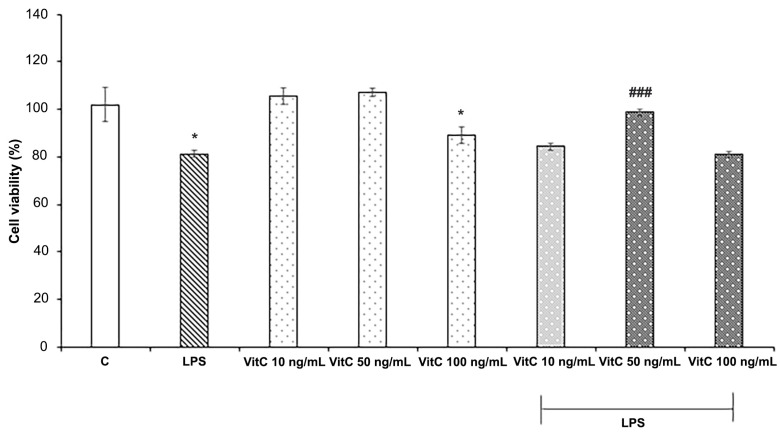
Cell viability analysis. Cells were treated in triplicate with different concentrations of Vit C, ranging from 10 to 100 ng/mL, alone or in combination with LPS (1 μg/mL). Untreated cells represent the control (C). Cell viability was evaluated after 48 h by the MTT assay. Results are expressed as the mean ± SD of 3 measurements (n = 5 experiments). The results are expressed in percentages with respect to the control (mean ± SD, * *p* < 0.05 vs. C; ### *p* < 0.001 vs. LPS).

To define the optimal Vit C concentration for in vitro experiments, the effects of Vit C and LPS on cell viability were evaluated by the MTT assay. For the purpose, three different concentrations of Vit C, ranging from 10 ng/mL to 100 ng/mL, alone or in combination with LPS (1 μg/mL) were tested, by evaluating the cell viability percentage with respect to controls (Figure 1), according to the experimental procedures previously described.

Data reported in Figure 1 show that LPS treatment determined a significant reduction in cell viability (*p* < 0.05) in comparison to the control; among the Vit C concentrations tested, both 10 ng/mL and 50 ng/mL did not exert cytotoxic effects on BV-2 cells, whereas Vit C 100 ng/mL treatment determined a reduction in cell viability in comparison to the control (*p* < 0.05). Interestingly, Vit C 50 ng/mL pre-treatment determined a significant (*p* < 0.001) increase in cell viability of BV2 stimulated with LPS in comparison to those stimulated with LPS alone; thus, the concentration of 50 ng/mL was selected for the in vitro treatments.

### 3.2. Vit C Regulation of Pro-Inflammatory Mediators

As reported in [11] (see also Figure S1), MPTP-treated mice exhibited a marked reduction in TH expression at both SNpc and CP levels, thus validating the efficacy of the mouse model of PD determined by the destruction of TH-positive neurons following MPTP treatment. In mice treated with MPTP that received Vit C, TH immunoreactivity was more intense compared to animals that received only MPTP, indicating that Vit C reduces dopaminergic neuron loss induced by MPTP [11]. Moreover, in this work, we also observed that the pre-treatment with Vit C determined, in different brain areas, a reduced expression of TLR4 and p-IKBα, two molecules involved in the NF-κB signaling pathway [11].

**Figure 2:**
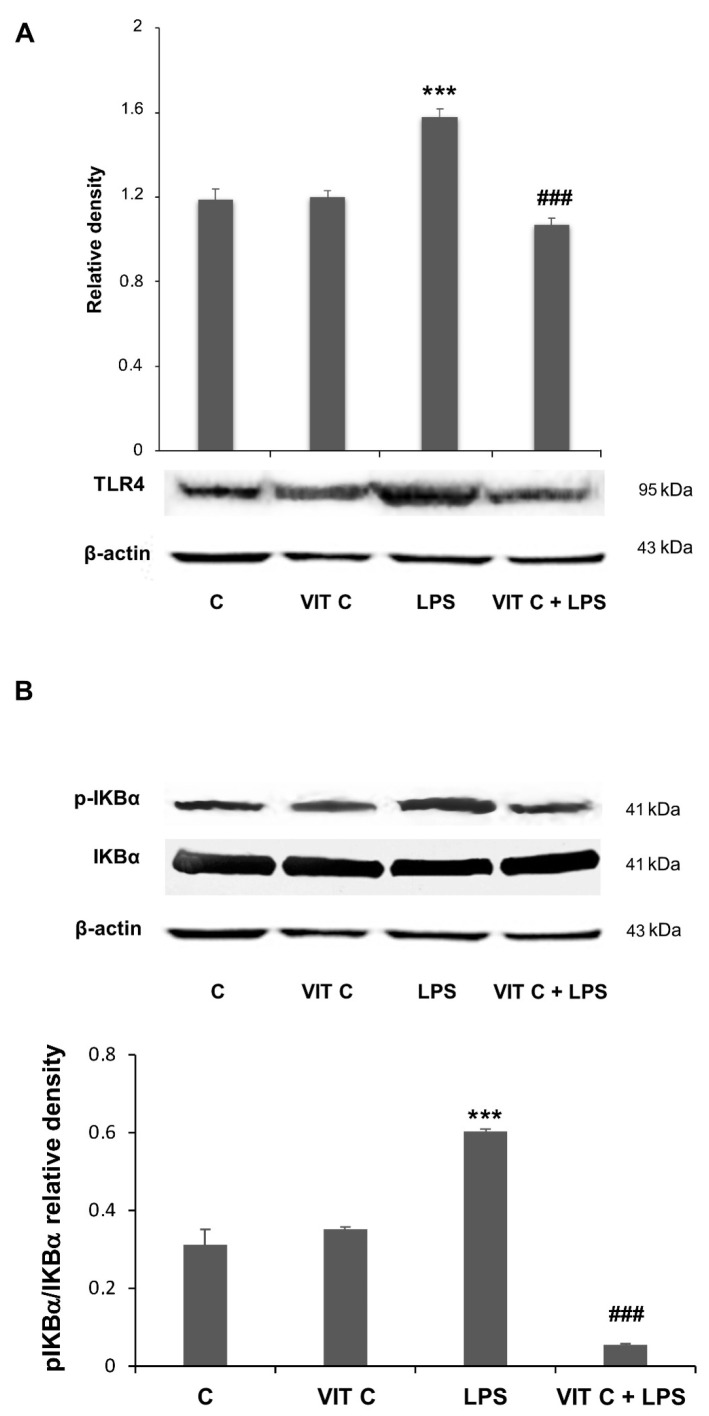
Vit C effects on pro-inflammatory mediators’ regulation. The expression levels of TLR4 (**A**) and the p-IKBα/IKBα (**B**) ratio were evaluated by a densitometric analysis of Western blots. The same membrane was used for the immunodetection of different proteins by the removal (stripping) of primary and secondary antibodies and then normalizing the bands against the same β-actin. Results are expressed as the mean ± SD of 3 measurements (n = 5 experiments); (*** *p* < 0.001 vs. C; ### *p* < 0.001 vs. LPS).

In the present work, we assessed if these mediators were subjected to a modulation in BV-2 cells submitted to Vit C treatment. The Western blotting analysis showed an increase in both TLR4 (Figure 2A) and the pIKBα/IKBα ratio (Figure 2B) in response to the LPS treatment. However, when LPS-stimulated cells were pre-treated with Vit C, the expression of these mediators was significantly reduced in comparison to treatment with LPS alone; these results are consistent with those obtained in the in vivo model [11].

**Figure 3:**
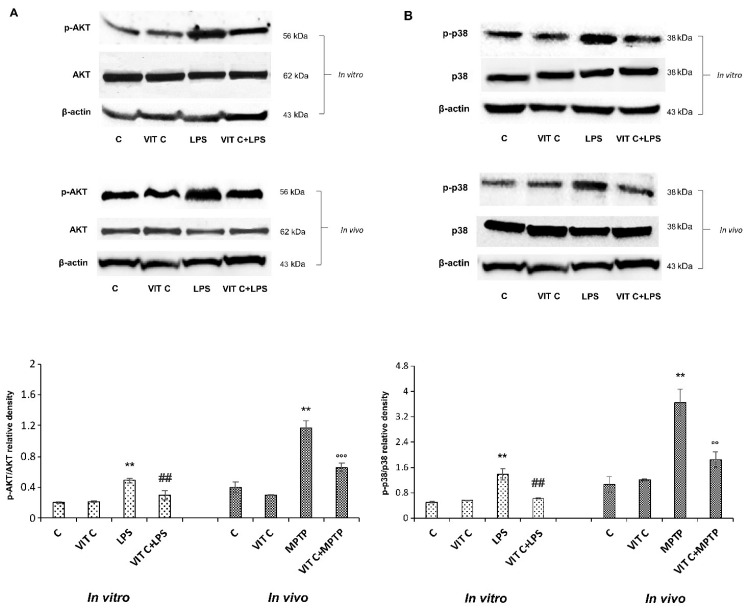
Expression analysis of p-AKT and p-p38. The expression levels and densitometric analysis of p-AKT/AKT (**A**) and the p-p38/p38 ratio (**B**) were evaluated on the same membrane by the removal (stripping) of primary and secondary antibodies and then normalizing the bands against the same β-actin. Results are expressed as the mean ± SD of 3 measurements (n = 5 experiments); (** *p* < 0.01 vs. C; ## *p* < 0.01 vs. LPS; °° *p* < 0.01 vs. MPTP; °°° *p* < 0.001 vs. MPTP).

To characterize the molecular mechanisms underlying Vit C effects, we considered the signaling pathways depending on TLR4 activation, playing a role in regulating inflammatory gene expression through the NF-κB transcription factor [13]. Therefore, we looked at the PI3K/AKT and the p38 MAPK signaling pathways, analyzing the protein expression of respective effectors p-AKT and p-p38 both in vitro and in vivo. Figure 3 reports the immunoblots of p-AKT and AKT (panel A, upper) and the densitometric analysis of their ratio (panel A, lower), and the immunoblots of p-p38 and p38 (panel B, upper) and the densitometric analysis of their ratio (panel B, lower).

LPS treatment determined both in BV-2 and in mice SNpc a significant upregulation of p-AKT as well as p-p38 expression in comparison to unstimulated cells. Moreover, results showed that Vit C pre-treatment before LPS stimulation significantly counteracted this upregulation both in BV-2 cells and in MPTP-treated mice, thus evidencing the capacity of Vit C to regulate MAPK and PI3K/AKT signaling pathways, both involved in the inflammatory responses.

### 3.3. Vit C Regulation of GSK3β

**Figure 4:**
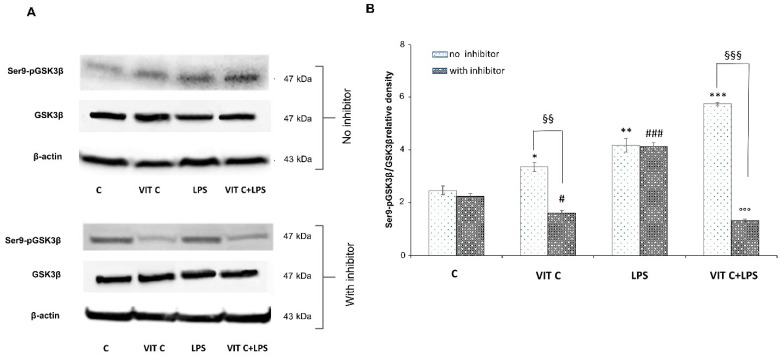
Expression analysis of Ser9p-GSK3β/GSK3β ratio in presence or in absence of SB203580 inhibitor. The expression levels of Ser9p-GSK3β/GSK3β (**A**) were evaluated by the Western blotting and densitometric analysis. β-actin was used for normalization (**B**). Results are expressed as the mean ± SD of 3 measurements (n = 5 experiments); (* *p* < 0.05 vs. C; ** *p* < 0.01 vs. C; *** *p* < 0.001 vs. C (no inhibitor); # *p* < 0.05 vs. C (with inhibitor); ### *p* < 0.001 vs. C (with inhibitor); §§ *p* < 0.01 (with inhibitor vs. no inhibitor); §§§ *p* < 0.001 (with inhibitor vs. no inhibitor); °°° *p* < 0.001 vs. LPS (no inhibitor)).

The above-mentioned pathways converge towards the GSK3β activation. Indeed, the active form of GSK3β is involved in the activation of the NF-κB transcription factor, while p-p38 MAPK and p-AKT are responsible for GSK3β inactivation, by phosphorylation at Ser9. Since GSK3β is considered a pivotal regulator of inflammatory and antioxidant responses, to elucidate its involvement in Vit C-mediated effects, we analyzed the protein expression levels of the anti-inflammatory Ser9p-GSK3β in BV-2 cells. For this purpose, we treated microglial cells with a selective p38 inhibitor, SB203580, to clarify the involvement of p38 MAPK in GSK3βSer9 phosphorylation. In Figure 4 panel A, immunoblots of Ser9p-GSK3β and the total form of GSK3β are reported, whereas the densitometric analysis of the Ser9p-GSK3β/GSK3β ratio is reported in panel B.

In Vit C-treated cells, we observed a significant increase (*p* < 0.05) in the active GSK3β in comparison to untreated cells, which resulted in a significant downregulation (*p* < 0.01) in the presence of the p38 inhibitor. Moreover, we proved that the p38 inhibition significantly counteracted (*p* < 0.01) the Ser9p-GSK3β upregulation in cells treated with Vit C alone compared to those without the inhibitor, suggesting the involvement of the p38 MAPK in GSK3β regulation. In cells treated with LPS alone, the p38 inhibitor did not reduce the expression of the inactive form of GSK3β in comparison to the results observed in LPS-treated cells without the inhibitor. In this regard, it is possible that the elevated levels of Ser9p-GSK3β observed in LPS-activated cells in the presence of the p38 inhibitor may be due to the increased levels of Akt that we previously observed. In fact, Ser9p-GSK3β upregulation by Akt has also been previously described [14]. Interestingly, Vit C treatment was able to increase Ser9p-GSK3β both alone and in the presence of LPS in comparison to controls, as reported in Figure 4. Finally, the inhibition of p38 in cells treated with Vit C+LPS significantly reduced (*p* < 0.001) the expression of the inactive form of GSK3β in comparison to that observed in the absence of the inhibitor; this is probably caused by the Vit C pre-treatment before LPS stimulation that counteracted the activation of Akt, as shown in Figure 2A. 

Overall, our results demonstrated that pre-treatment with Vit C was able to upregulate the Ser9p-GSK3β form and that this increase was presumably mediated by p38 protein.

**Figure 5:**
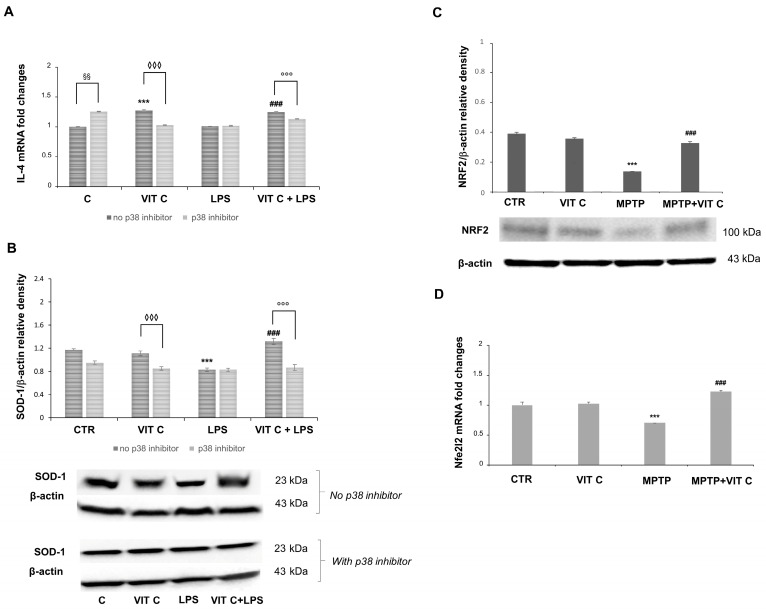
The Vit C-mediated anti-inflammatory and antioxidant responses. The in vitro IL-4 mRNA expression (**A**) was tested with end-point PCR and the subsequent densitometric analysis. The results were normalized vs. GAPDH. Results are expressed as the mean ± SD of 3 measurements (n = 5 experiments); (*** *p* < 0.001 vs. C, ### *p* < 0.001 vs. LPS; ◊◊◊ *p* < 0.001 vs. Vit C, no p38 inhibitor; §§ *p* < 0.01 vs. C, no p38 inhibitor; °°° *p* < 0.001 vs. VIT C+LPS, no p38 inhibitor). (**B**) In vitro expression levels of SOD-1 evaluated by the Western blotting and densitometric analysis. β-actin was used for normalization. Results are expressed as the mean ± SD of 3 measurements (n = 5 experiments); (*** *p* < 0.001 vs. C; ### *p* < 0.001 vs. LPS; °°° *p* < 0.001 vs. VIT C +LPS, no p38 inhibitor; ◊◊◊ *p* < 0.001 vs. Vit C, no p38 inhibitor). (**C**) In vivo expression levels of NRF2 evaluated by the Western blotting and densitometric analysis. β-actin was used for normalization. Results are expressed as the mean ± SD of 3 measurements (n = 5 experiments); (*** *p* < 0.001 vs. C, ### *p* < 0.001 vs. MPTP). (**D**) Nfe2l2 in vivo mRNA expression tested with end-point PCR and the subsequent densitometric analysis. The results were normalized vs. GAPDH. Results are expressed as the mean ± SD of 3 measurements (n = 5 experiments); (*** *p* < 0.001 vs. C, ### *p* < 0.001 vs. MPTP).

Since we previously evidenced in a PD mouse model [11] that Vit C treatment determined both the downregulation of the pro-inflammatory mediators and upregulation of the anti-inflammatory markers, in the present work, we also tested the anti-inflammatory and antioxidant effects of Vit C on microglial cells. In this respect, to elucidate the mechanism by which Vit C is responsible for its anti-inflammatory action, we evaluated the mRNA expression of the anti-inflammatory cytokine interleukin 4 (IL-4) and the protein expression of the free radical scavenging enzyme superoxide dismutase 1 (SOD-1), in the presence or in the absence of the p38 inhibitor (Figure 5).

A significant increase (*p* < 0.001) in IL-4 mRNA was observed in Vit C-treated cells in comparison to controls. IL-4 mRNA revealed more expression (*p* < 0.001) in Vit C+LPS-treated cells in comparison to cells treated with LPS alone. Following the p38 inhibition, the increase in IL-4 expression was significantly reduced both in cells treated with Vit C alone and in those stimulated with Vit C plus LPS, differently from what was observed in the same experimental conditions without the p38 inhibitor (Figure 5A). Also, in this case, LPS-activated cells exhibited the same levels of IL-4 mRNA both in the presence and in the absence of the p38 inhibitor. Overall, these results seem to suggest that the over-expression of this anti-inflammatory cytokine is probably due to the Vit C presence and that this effect may be mediated by p38 MAPK signaling. Surprisingly, we also detected a significant increase (*p* < 0.01) in untreated cells in the presence of the p38 inhibitor in comparison to controls without the p38 inhibitor. 

Concerning the SOD-1 expression, this enzyme resulted in the upregulation (*p* < 0.001) in LPS-treated cells after Vit C pre-treatment compared to cells stimulated with LPS alone, thus evidencing that Vit C was able to increase the antioxidant capacity of the cells. Interestingly, the p38 inhibition led to a significant reduction in the SOD-1 expression in comparison to the same treatments of cells carried out without the inhibitor, suggesting a possible modulation through p38 also for the antioxidant responses (Figure 5B) elicited by Vit C. Finally, we observed that Vit C significantly increased the expression, both at mRNA and at protein levels, of the antioxidant enzyme NRF2 in MPTP animals treated with Vit C in comparison to MPTP ones that did not receive Vit C, thus validating the GSK3 role regarding SOD-1 regulation (Figure 5C,D).

### 3.4. Vit C Effects on Microglial Phenotype

**Figure 6:**
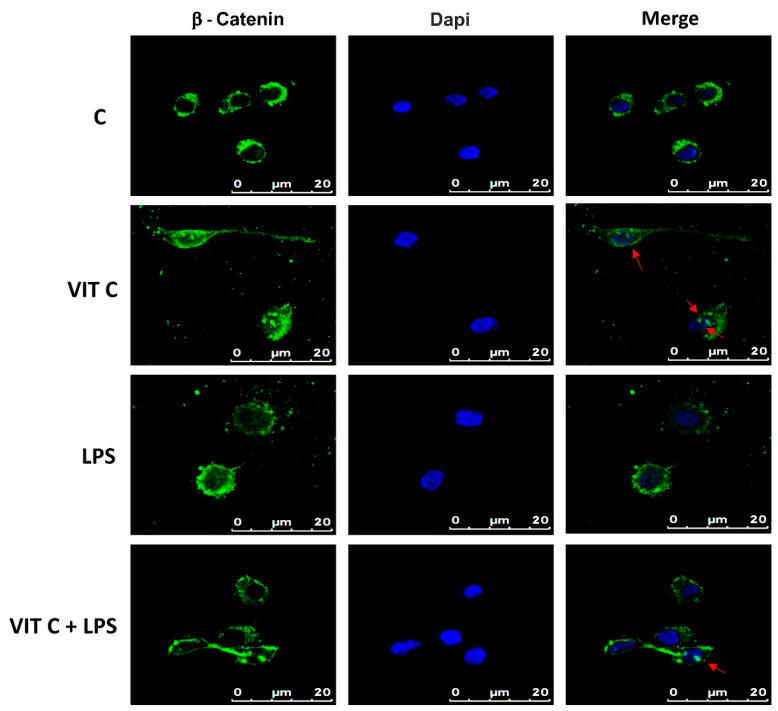
Cytolocalization of β-catenin. Fluorescent images with FITC-stained β-catenin (green) and DAPI-stained nuclei (blue) were obtained by merging images. Results are representative of 5 different experiments; scale bars: 20 μm. Red arrows indicate the presence of β-catenin inside the nuclei.

Among the targets of GSK3β is β-catenin, which plays a role in the inflammatory response regulation, acting as an NF-κB inhibitor, thus influencing the microglial phenotype [8]. β-catenin is normally directed to proteasomal degradation through the GSK3β-mediated phosphorylation. In contrast, the GSK3β inactivation leads the cytoplasmic β-catenin towards the subsequent nuclear translocation. Therefore, we assayed the intracellular localization of β-catenin in BV-2 cells by immunofluorescence (Figure 6).

Immunofluorescence images showed that while in the control and in LPS-treated cells, β-catenin is mainly localized in perinuclear space, and in cells treated with Vit C alone or in combination with LPS, a nuclear translocation of β-catenin was observed, as demonstrated by the presence of globular staining inside the nuclei, indicated by red arrows (Figure 6). These results suggest that GSK3β inhibition could attenuate the activation of the NF-κB-mediated pro-inflammatory responses and promote the anti-inflammatory functions.

**Figure 7:**
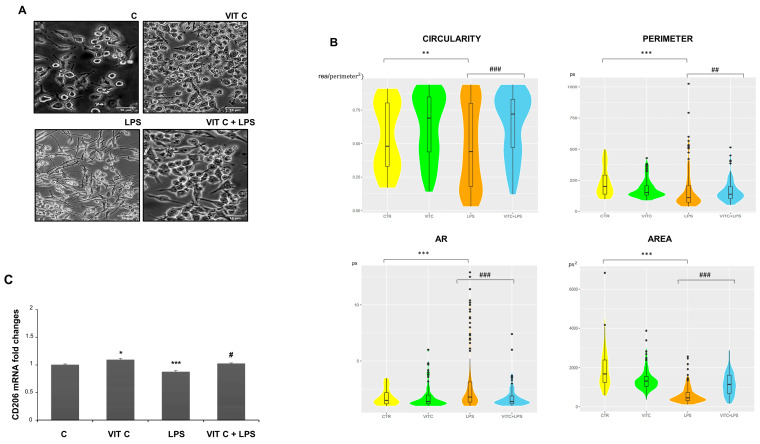
Morphometric analysis of BV2 cells. Cell images (**A**) were taken with a 200X objective magnification with brightfield microscopy. Scale bars: 10 μm. Morphometric analyses (**B**) were performed with the ImageJ software considering the circularity, perimeter, AR, and area. The data are represented with a violin graph. (**C**) CD206 mRNA expression with end-point PCR and subsequent densitometric analysis. Results are expressed as the mean ± SD of 3 measurements (n = 5 experiments). (* *p* < 0.05 vs. C; ** *p* < 0.01 vs. C; *** *p* < 0.001 vs. C; # *p* < 0.05 vs. LPS; ## *p* < 0.01 vs. LPS; ### *p* < 0.001 vs. LPS.)

In addition, we examined the morphology of microglial cells through a morphometric analysis by measuring the area, perimeter, circularity, and aspect ratio (AR) (Figure 7). The perimeter is defined as the sum of the individual distances between adjacent points of the shape outline, the area is the number of pixels (px) within the shape, circularity is the ratio between the area and the square of the perimeter, and, lastly, the AR refers to the ratio of the cellular major axis to the minor axis [15].

The morphometric analysis (Figure 7B) showed that BV-2 cell morphology is influenced by Vit C treatment. In particular, Vit C promoted the enlargement of soma with a subsequent expansion of size, leading the cells to assume a round shape. On the contrary, the LPS-stimulated cells showed an elongated morphology characterized by a reduction in the area and perimeter in comparison to the control. To clarify if the morphology showed by Vit C-pre-treated cells was associated with the anti-inflammatory M2 phenotype, we analyzed the mRNA expression of the M2 phenotype marker CD206 by RT-PCR. As observed, the CD206 expression showed that this receptor was more expressed in microglial cells treated with Vit C alone in comparison to the control, as well as in Vit C-pre-treated cells plus LPS in comparison to the cells stimulated with LPS alone, suggesting that the Vit C treatment was able to upregulate the anti-inflammatory M2 phenotype (Figure 7C).

## 4. Discussion

Many vitamins are well known for their anti-inflammatory and antioxidant properties; thus, vitamin supplementation has been suggested as valuable support in the therapeutic strategy for neuroinflammatory disease treatment. In this respect, our research group has investigated in previous studies the neuroprotective effects of different vitamins, including Vit D, Vit C, and Vit E [11,16,17], confirming their neuroprotective role. Vit C is involved in CNS homeostasis, entering the CNS through the choroid plexus epithelium via type 2 sodium-dependent transporters (SVCT2), passing from plasma to the cerebrospinal fluid. Vit C can also enter the CNS in its oxidized form, the dehydroascorbate, via the glucose transporter GLUT1 present in the blood–brain barrier endothelium [18]. Once in the cerebrospinal fluid, neurons take ascorbate via SVCT2 and dehydroascorbate via GLUT1, while glial cells uptake only dehydroascorbate via GLUT1 [19]. Although the Vit C anti-inflammatory and antioxidant properties have been demonstrated in different animal models, such as MPTP- and LPS-treated mice, or in colchicine- and ethanol-treated rats [11,20,21,22], in actuality, there are no studies clarifying the intracellular signaling pathway by which Vit C is able to play its protective role during neuroinflammation, which may, in turn, underlie neurodegeneration. In this respect, in this work, we explored both in a well-consolidated MPTP mouse model of PD, in which we have already detected a reduction in pro-inflammatory mediators induced by Vit C treatment [11], and in an in vitro model of neuroinflammation, represented by LPS-induced BV-2 cells, the molecular mechanism underlying the Vit C effects on microglial cells. We demonstrated the ability of Vit C to decrease the expression of different mediators involved in the inflammatory responses, such as TLR4, p-IKBα, and the phosphorylated forms of p38 and AKT. It is well known that LPS through TLR4 is able to activate MAPK, AKT, and NF-κB, inducing the release of pro-inflammatory cytokines; in addition, the MPTP neurotoxin triggers the release of many inflammatory mediators [23,24]. Thus, the downregulation of these mediators by Vit C observed in our experimental models proved its ability to attenuate neuroinflammation by regulating crucial mediators, including TLR4, MAPK, and PI3K/AKT signaling pathways. We focused on GSK3β, downstream-regulated by the mentioned pathways, that is involved in the regulation of many inflammatory responses. In particular, its activated state is associated with a pro-inflammatory response via the activation of NF-κB; the inactivated GSK3β leads to an anti-inflammatory response by inactivating NF-κB and activating the cAMP-response element binding protein (CREB), activator protein 1 (AP-1), the signal transducer and activator of transcription 1–3 (STAT1–3), nuclear factor erythroid 2-related factor 2 (NRF2), and β-catenin [8]. The defective inhibition of GSK3β by Ser9 phosphorylation has been related to inflammatory neuropathologies, such as PD [25]. 

Phosphorylation is a regulatory mechanism of GSK3 activity with major implications in the control of the immune response. Thus, GSK3 inhibition by classical Ser9 phosphorylation upregulates the production of anti-inflammatory mediators and downregulates the release of pro-inflammatory cytokines [26], although it is also involved in the resolution of inflammation in an in vivo model, as reported in study [27]. Within the brain, the defective inhibition of GSK3β by Ser9 phosphorylation has been related to inflammatory neuropathologies, including PD [28].

p38 MAPK regulates the phosphorylation of GSK3β at Ser9 and the dephosphorylation of GSK3β Thr389, mechanisms that appear to play a significant role in autophagy and cell death [29]. 

Conversely, GSK3 phosphorylation at Ser-9 is reported to be involved in inflammatory responses, as reported in literature [30,31].

Indeed, increased GSK3β levels have been reported in peripheral blood lymphocytes in patients with PD [32]. In addition, in an MPTP mouse model of PD, the inhibition of GSK3β protected against MPTP toxicity and decreased α-synuclein protein expression [33,34]. Here, we demonstrated that Vit C promotes the inhibition of GSK3β by increasing the expression of the GSK3β inactivated form, leading to antioxidant and anti-inflammatory effects: these may be mediated, at least in part, by the p38 pathway since p38 inhibition led to a decrease in Ser9p-GSK3β and a downregulation of the IL-4 cytokine expression. In this context, under unstimulated conditions, anti-inflammatory responses could be observed as a result of p38 inhibition, because cells, to compensate this inhibition, may activate other signaling pathways, such as PKA/AMPK. The slight IL-4 upregulation observed in controls could therefore be attributable to an effect exerted through the activation of these pathways as reported by other observations [35,36]. In addition, Vit C pre-treatment markedly increased SOD expression, which resulted, also in this case, in the downregulation in the presence of the p38 inhibitor. Overall, the SOD upregulation by Vit C treatment may be associated with neuroprotective effects as previously reported by other authors [20].

Interestingly, we observed the upregulation of NRF2 in MPTP animals previously treated with Vit C in comparison to MPTP-only-treated ones. In this regard, NRF2 is an antioxidant enzyme, which can inhibit oxidative stress: it has been shown that in vivo, it possesses anti-inflammatory effects, inhibiting the release of inflammatory factors such as TNF-α, IL-1β, and MIP-1, and counteracting oxidative stress [37,38,39]. Therefore, the increased expression of NRF2 in our PD in vivo model confirms that Vit C promotes anti-inflammatory responses.

GSK3β plays a key role in regulating the Wnt/β-catenin signaling pathway whose effector, β-catenin, not only acts as an NF-κB inhibitor but also plays a role in determining the microglial phenotype [40]. GSK3β, forming a multimeric complex with Axin and APC, normally is responsible for β-catenin phosphorylation and its subsequent proteasomal degradation. When the GSK3β is inactivated, β-catenin accumulates within the cytoplasm and then moves inside the nuclei to work as a transcription factor [41]. In this respect, GSK3β inhibition has also been shown to induce the secretion of the anti-inflammatory cytokine IL-10 [42]. The downregulation of the Wnt canonical pathway has been correlated with dopaminergic neuron degeneration in vitro [42,43] and neuronal loss has been linked to increased GSK3β activity other than lower β-catenin levels both in in vivo and in vitro models of PD [44]. Our β-catenin cytolocalization studies showed that the treatment with Vit C leads to β-catenin translocation inside the nuclei, evidencing a Wnt/β-catenin signaling pathway activation towards the control of inflammation. In addition, the morphometric analysis of BV-2 cells demonstrated that LPS promoted a rod-shape morphology characterized by a spindle-like soma and elongated processes after 48 h of incubation, which has been described as a transitional morphology of microglia able to quickly reply to LPS insult eliciting pro-inflammatory responses [45]. The pre-treatment with Vit C, instead, promoted round morphology and hypertrophic microglia characterized by an enhanced M2 phenotype, as demonstrated by the CD206 upregulation, suggesting that Vit C drives the microglia defensive functions by supporting the M2 phenotype. 

## 5. Conclusions

The results of this study showed that the anti-inflammatory and the antioxidant functions of Vit C may be dependent on the GSK3β inhibition, promoting the M2 phenotype and involving the Wnt/β-catenin signaling pathway, both in an in vivo and in an in vitro experimental model. To better clarify the mechanisms by which Vit C carries out its protective effects, further studies with different approaches, such as using silent cell models and/or additional inhibitors, are needed. Overall, our observations suggest that Vit C administration may represent a potential strategy to control neuroinflammation, opening possible future scenarios for therapeutic use of Vit C in neurodegenerative disease treatment.

## Figures and Tables

**Table 1:** List of primers used for RT-PCR.

cDNA Target	Sequence (5′–>3′)	Sequence References
IL4	FW: CTCCTAGCAACCACGGCCCC RW: GCTAGGCATAACGCACTAGGTT	NM_021283.2
GAPDH	FW: ACCACAGTCCCTGCCATCAG RW: TCCACCACCCTGTTGCTGTA	NM_001411840.1
CD206	FW: AACCAGTTCCTTGAGCTCGG RW: CTGATTAGGGCAGCCGGTAG	NM_008625.2
NRF2	FW: CAAGACTTGGGCCACTTAAAAGAC RW: AGTAAGGCTTTCCATCCTCATCAC	XM_021193142.2

## Data Availability

The original contributions presented in the study are included in the article/Supplementary Materials, further inquiries can be directed to the corresponding author.

## Supplementary Materials

The following supporting information can be downloaded at: https://www.mdpi.com/article/10.3390/nu16081121/s1, Figure S1: Tyrosine hydroxylase (TH) analysis. TH immunoreactive catecholaminergic neurons of SNpc and their projections in the striatum (upper) and SNpc (lower) in controls (CTR), mice treated with Vitamin C (VIT C), MPTP, MPTP and Vitamin C (MPTP+ VIT C). Scale bar: (striatum) upper 1000 μm (objective 4×), lower 50 μm (obj. 40×); (SNpc) upper 500 μm (obj. 4×), lower 100 μm (obj. 20×).
