# Supplementary material for: Ser9p-GSK3β Modulation Contributes to the Protective Effects of Vitamin C in Neuroinflammation
Source: Nutrients. 2024 Apr 10;16(8):1121. doi: 10.3390/nu16081121 (PMC11053771; doi:10.3390/nu16081121)
Supplement: Supplementary file 1 [file nutrients-16-01121-s001.zip › nutrients-2900552-supplementary.pdf]

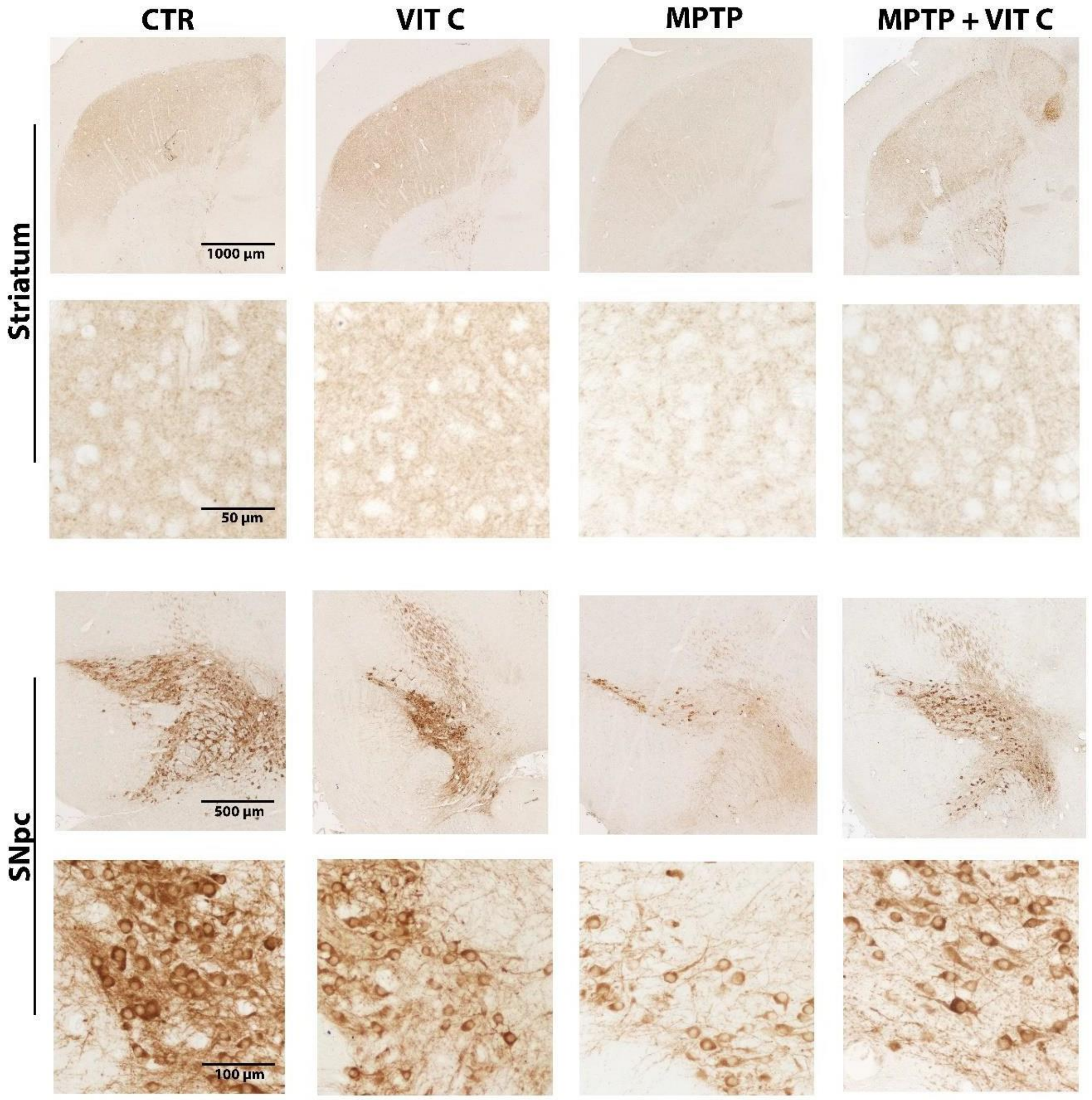

Supplementary figure S1

Tyrosine hydroxylase (TH) analysis. (A) TH immunoreactive catecholaminergic neurons of SNpc and their projections in the striatum (upper) and SNpc (lower) in controls (CTR), mice treated with Vitamin C (VIT C), MPTP, MPTP and Vitamin C (MPTP+ VIT C). Scale bar: (striatum) upper 1000 µm (objective 4×), lower 50 µm (obj. 40×); (SNpc) upper 500 µm (obj. 4×), lower 100 µm (obj. 20×).
